# Mice Transgenic for CD4-Specific Human CD4, CCR5 and Cyclin T1 Expression: A New Model for Investigating HIV-1 Transmission and Treatment Efficacy

**DOI:** 10.1371/journal.pone.0063537

**Published:** 2013-05-15

**Authors:** Kieran Seay, Xiaohua Qi, Jian Hua Zheng, Cong Zhang, Ken Chen, Monica Dutta, Kathryn Deneroff, Christina Ochsenbauer, John C. Kappes, Dan R. Littman, Harris Goldstein

**Affiliations:** 1 Department of Microbiology & Immunology, Albert Einstein College of Medicine, Bronx, New York, United States of America; 2 Department of Pediatrics, Albert Einstein College of Medicine, Bronx, New York, United States of America; 3 Department of Developmental and Molecular Biology, Albert Einstein College of Medicine, Bronx, New York, United States of America; 4 Department of Medicine, University of Alabama at Birmingham, Birmingham, Alabama, United States of America; 5 Birmingham Veterans Affairs Medical Center, Research Service, Birmingham, Alabama, United States of America; 6 Molecular Pathogenesis Program, Skirball Institute of Biomolecular Medicine, New York University School of Medicine, New York, New York, United States of America; 7 Howard Hughes Medical Institute, Skirball Institute of Biomolecular Medicine, New York University School of Medicine, New York, New York, United States of America; George Mason University, United States of America

## Abstract

Mice cannot be used to evaluate HIV-1 therapeutics and vaccines because they are not infectible by HIV-1 due to structural differences between several human and mouse proteins required for HIV-1 entry and replication including CD4, CCR5 and cyclin T1. We overcame this limitation by constructing mice with CD4 enhancer/promoter-regulated human CD4, CCR5 and cyclin T1 genes integrated as tightly linked transgenes (hCD4/R5/cT1 mice) promoting their efficient co-transmission and enabling the murine CD4-expressing cells to support HIV-1 entry and Tat-mediated LTR transcription. All of the hCD4/R5/cT1 mice developed disseminated infection of tissues that included the spleen, small intestine, lymph nodes and lungs after intravenous injection with an HIV-1 infectious molecular clone (HIV-IMC) expressing *Renilla reniformis* luciferase (LucR). Furthermore, localized infection of cervical-vaginal mucosal leukocytes developed after intravaginal inoculation of hCD4/R5/cT1 mice with the LucR-expressing HIV-IMC. hCD4/R5/cT1 mice reproducibly developed in vivo infection after inoculation with LucR-expressing HIV-IMC which could be bioluminescently quantified and visualized with a high sensitivity and specificity which enabled them to be used to evaluate the efficacy of HIV-1 therapeutics. Treatment with highly active anti-retroviral therapy or one dose of VRC01, a broadly neutralizing anti-HIV-1 antibody, almost completed inhibited acute systemic HIV-1 infection of the hCD4/R5/cT1 mice. hCD4/R5/cT1 mice could also be used to evaluate the capacity of therapies delivered by gene therapy to inhibit in vivo HIV infection. VRC01 secreted in vivo by primary B cells transduced with a VRC01-encoding lentivirus transplanted into hCD4/R5/cT1 mice markedly inhibited infection after intravenous challenge with LucR-expressing HIV-IMC. The reproducible infection of CD4/R5/cT1 mice with LucR-expressing HIV-IMC after intravenous or mucosal inoculation combined with the availability of LucR-expressing HIV-IMC expressing transmitted/founder and clade A/E and C Envs will provide researchers with a highly accessible pre-clinical in vivo HIV-1-infection model to study HIV-1 acquisition, treatment, and prevention.

## Introduction

Two major restrictions prevent HIV-1 from infecting mouse cells. First, HIV-1 is unable to enter mouse cells because its envelope glycoprotein, gp120, does not engage mouse CD4 and CCR5 [1]. Second, HIV-1 Tat does not function in mouse cells because it does not bind to mouse cyclin T1 and consequently cannot activate HIV-1 transcription by recruiting the positive transcription elongation factor b (P-TEFb) complex to the HIV-1 TAR RNA target element [2]–[4]. To circumvent this restriction, humanized mouse models have been developed and used for HIV-1 investigation such as severe combined immunodeficient (SCID) mice transplanted with human peripheral blood lymphocytes [5] or implanted with human fetal thymus and liver [6], Rag2^−/−^γ_c_^−/−^ mice injected with human hematopoietic stem cells (hHSC) [7], [8], NOD/SCID/IL2Rγ^null^ mice injected with hHSC [9] or NOD/SCID mice transplanted with human fetal thymus and liver tissue and injected with syngeneic hHSC [10]. However, these humanized mouse models cannot take advantage of the wide array of available transgenic and gene-deleted mouse lines to apply genetic approaches to investigate HIV-1 transmission. Their construction is also technically challenging, time-consuming and expensive. They do not generate potent HIV-1-specific human immune responses which limit their usefulness for evaluating HIV-1 vaccines and HIV-1 immunopathogenesis.

Transgenic mice have been generated to overcome these restrictions by crossing transgenic lines carrying CD4 promoter/enhancer cassettes that direct expression of human CD4, CCR5 or cyclin T1 transgenes to CD4 T lymphocytes, macrophages, and monocytes. However, productive in vivo infection in these transgenic mice has not been reported [11]. Two limitations have prevented their use for in vivo HIV-1 infection studies. First, the time-consuming and inefficient process of breeding three separate lines transgenic for human CD4, CCR5 or cyclinT1 impedes the generation of sufficient mice for experiments because only one of eight progeny mice are predicted to carry all three alleles after a heterozygous cross. Second, clearly demonstrating productive in vivo HIV-1 infection is complicated by the absence of a highly sensitive and specific method of monitoring HIV-1 replication in the context of the reduced capacity of mice to support efficient HIV-1 replication. We overcame both of these limitations by generating an improved mouse model carrying the human CD4, CCR5 and cyclin T1 transgenes transmitted as a single allele that is co-inherited across multiple generations with targeted expression to CD4+ T cells and macrophages (hCD4/R5/cT1 mice) and using a recently developed replication-competent molecular HIV-1 clone that expresses *Renilla reniformis* luciferase (LucR) as the infectious inoculum [12].

## Materials and Methods

### Construction of Transgenic Mice

**Figure 1:**
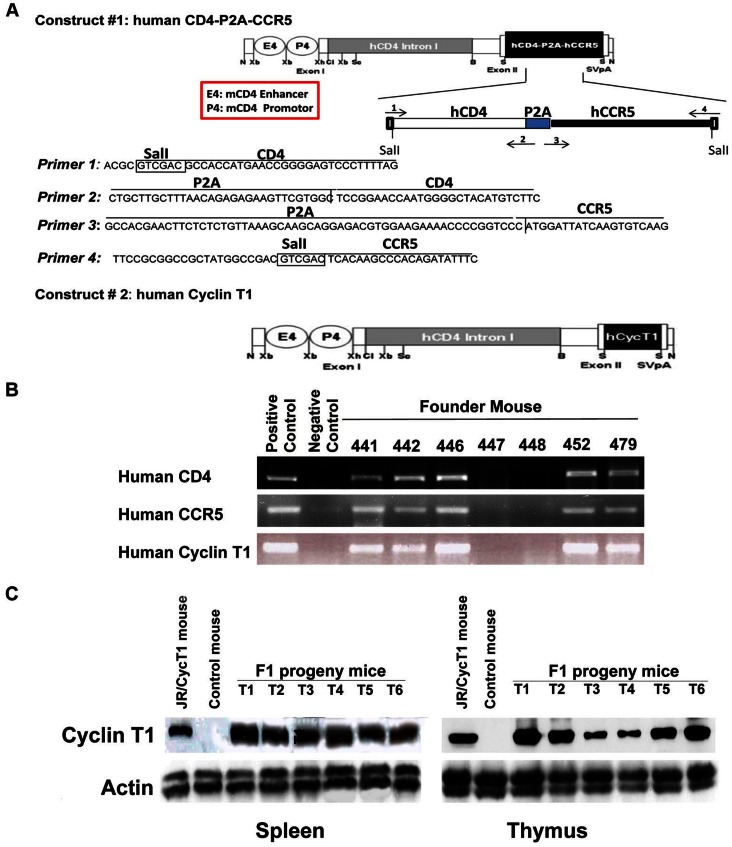
Construction of hCD4/R5/cT1 mice and evaluation of transgene expression. (**A**) Schematic representation of the human CD4/CCR5 and cyclin T1 transgene constructs. E4/P4, murine CD4 enhancer/promoter; N, Xb, Xh, Cl, Sc, B, and S, restriction enzyme sites for NotI, XbaI, Xho1, ClaI, SacI, BamHI and SalI, respectively; SVpA, SV40 polyadenylation signal. (**B**) Transmission of the human CD4 and CCR5 or cyclin T1 in the transgenic mouse founders was determined by PCR. DNA was extracted from the tails of the indicated transgenic founder mice and integrated human CD4, CCR5 or cyclin T1 genes were detected by PCR amplification with primer pairs specific for human CD4, CCR5 and cyclin T1, respectively. (**C**) Expression of the human cyclin T1 transgene in the spleens and thymuses of founder 479 progeny mice was detected by immunoblotting with a polyclonal antibody specific for human cyclin T1. Lysate from spleens from a JRCSF/hu-CycT1 mouse and a wild-type littermate mice were run as positive and negative controls respectively and equivalent protein loading was demonstrated by immunoblotting with a polyclonal antibody specific for mouse actin.

A vector expressing human CD4 and CCR5 as a single transcript with the genes linked by a self-cleaving picornovirus-derived 2A peptide sequence was constructed using the approach we previously described [13]. Full-length human CD4 and CCR5 genes were cloned by PCR amplification using the pT4B and pCCR-5 vectors (obtained through the NIH AIDS Research and Reference Reagent Program, from Dr. Richard Axel and Dr. Nathaniel Landau, respectively) [1], [14], [15] as templates for the human CD4 and CCR5 genes, respectively, and were combined into a single sequence linked by the 2A sequence (CD4-2A-CCR5) using a modification of a previously described approach [13]. Briefly, as shown in Figure 1A, the human CD4 gene was amplified by PCR with a primers specific for the 5′ leader sequence of the CD4 with an added Sal I restriction site (primer 1) and for the 2A sequence followed by the terminal CD4 region (primer 2). The human CCR5 gene was amplified by PCR with primers specific for the full 2A sequence followed by the CCR5 leader sequence (primer 3) and the terminal sequence of the CCR5 constant region and an added Sal I restriction site (primer 4). The two PCR products were mixed and the human CD4-2A-CCR5 sequence was generated by PCR amplification with primers 1 and 4 and cloned into the Sal I restriction site of the CD4 promoter/enhancer cassette-regulated vector previously used to construct the CD4 promoter/enhancer-regulated human cyclin T1 vector [16]. Transgenic hCD4/R5/cT1 mouse founders were generated by microinjecting the human CD4-2A-CCR5 and human cyclinT1 constructs together into fertilized oocytes from C57BL/6 mice after digestion and excision of the inserts with NotI. Founders carrying the human CD4, CCR5, and cyclin T1 transgenes were identified by PCR analysis from tail DNA samples using human-specific internal primer sets for CD4 (5′ primer: GTGGAGTTCAAAATAGACATCGTG, 3′ primer: CAGCACCCACACCGCCTTCTCCCGCTT), CCR5 (5′ primer: CACCTGCAGCTCTCATTTTCC, 3′ primer: TTGTAGGGAGCCCAGAAGAG) and cyclin T1 (5′ primer: TCCCAACTTCCAGTTGGTACT, 3′ primer: TCCACCAGACCGAGGATTCAG). Founders carrying all three transgenes were mated with C57BL/6 mice, and transgene transmission to their progeny was determined by PCR amplification of tail DNA using the human CD4, CCR5 and cyclin T1 specific primers described above. Expression of human CD4 and CCR5 and expression of human cyclin T1 was determined by flow cytometry and immunoblot analysis, respectively, as described below.

### Flow Cytometric Analysis

Bone marrow cells, thymocytes, peripheral blood mononuclear cells (PBMC) and splenocytes isolated from the mice stained with phycoerythrin (PE)-labeled anti-human CCR5, Cy7-allophycocyanin (Cy7-APC)-labeled anti-human CD4 (Biolegend, San Diego, CA), and either FITC-labeled anti-mouse CD4 or FITC-labeled anti-mouse CD8 were analyzed for expression of the surface molecules using an LSRII (BD Biosciences, San Jose, CA) and FlowJo software (Treestar, Ashland, OR). For analysis of the phenotype of HIV-1-infected cells, we utilized NLENG1i-Bal.ecto or NLENG1i-CH077.ecto [17]. These replication-competent, *nef*-expressing HIV-1 infectious molecular clones were derived from the *gfp*-expressing NLENG1-IRES clone [18]–[20] modified to encode either the HIV-1 BaL *env* sequence or the CH077 *env* sequence, which was derived from a transmitted/founder HIV-1 isolate [12]. HIV-1-infected cells were identified by their expression of GFP and phenotypically characterized by staining with PE-labeled anti-mouse CD4 or APC-labeled anti-mouse CD11c.

### Immunoblot Detection of Human Cyclin T1

Human cyclin T1 was detected in whole cell extracts of mouse splenocytes or thymocytes by immunoblot as described [16]. Briefly, cellular lysate (25 µg) was resolved on a 10% sodium dodecyl sulfate-polyacrylamide gel electrophoresis gel and transferred to a nitrocellulose membrane (GE Water & Process Tech, Treveose, PA). The membrane was blocked for 1 hour with PBS containing 5% milk powder, then sequentially incubated either with goat anti-human cyclin T1 antibody or with goat anti-mouse actin antibody (Santa Cruz Biotechnologies, Santa Cruz, CA) and then with alkaline phosphatase-conjugated anti-goat IgG antibody. Bound antibody was detected with the Western Lightning chemiluminescence system (GE Healthcare, Boston, MA) according to the manufacturer’s instructions.

### Generation of HIV-1 Infectious Molecular Clones

Virus stocks from an HIV-1 infectious molecular clone encoding the *Renilla reniformis* luciferase gene was generated by transient transfection of 293T cells with NL-LucR.T2A-BaL.ecto plasmid DNA which encodes an HIV-1 provirus and a full-length Env protein in which the ectodomain sequence is derived from the BaL env, as previously described [12], [21]. The infectious titer of the NL-LucR.T2A-BaL.ecto virus (5–10×10^7^ infectious units (IU)/ml) was determined by limiting dilution infection of TZM-bl cells as described [12]. Virus stocks for the infectious molecular clones NLENG1i-BaL.ecto and NLENG1i-CHO77.ecto expressing GFP (see above) were derived using the same transfection and titration methods.

### Isolation of Mouse Myeloid-lineage Cells and CD4+ T Lymphocytes

Mouse femurs were lavaged with PBS containing 3% BSA to extract bone marrow cells which were dispersed by vigorous pipetting, washed, and cultured in complete media [RPMI media containing 10% FCS and 2-mercaptoethanol (50 µM)]. After 5 days of culture, nonadherent cells were gently rinsed away, fresh complete media chilled to 4°C was added, and adherent cells were harvested by scraping. These myeloid-lineage cells were greater than 95% viable by trypan blue exclusion and greater than 80% of the cells expressed the monocyte marker, CD11b, as detected by flow cytometric analysis. CD4+ T lymphocytes were purified from mouse splenocytes using the AutoMACS system (Miltenyi Biotec, Auburn, CA) in accordance with the manufacturer’s protocol. After lysis of red blood cells in the spleen by incubation in NH_4_Cl_2_ buffer (150 mM), mononuclear cells were washed twice with PBS, incubated with MACS MicroBeads coupled to anti-mouse CD4 antibody and passed through a positive selection autoMACS separation column using the AutoMACS automated bench-top magnetic cell sorter. Human CD4+ T cells or monocytes were isolated from PBMCs obtained from a HIV-1-naïve donor by immunomagnetic sorting using MACS MicroBeads coupled to anti-human CD4 or anti-human CD11b antibody, respectively, and passage through the AutoMACS automated bench-top magnetic cell sorter as described above. The purity of the sorted cells was determined by flow cytometry and was greater than 80%.

### HIV-1 Infection

Mouse myeloid-lineage cells or human macrophages isolated as described above were plated in 48 well plates (10^5^ cells/well) with complete media containing mouse or human GM-CSF (20 ng/ml). Highly purified mouse or human CD4+ T cells, isolated as described above, were stimulated with soluble anti-CD3 (3 µg/ml) and anti-CD28 (1 µg/ml) for 3 days and plated in 48 well plates (10^5^ cells/well) with complete media and added IL-2 (100 units/ml). The cells were spinfected in triplicate or duplicate cultures with NL-LucR-T2A-BaL.ecto by centrifugation in the presence of polybrene (4 µg/ml) at 2,500 RPM for 60 minutes. The virus was then washed away and the cells were placed in culture. At the indicated time, cells were harvested, and LucR activity was measured in duplicate using the Renilla Luciferase Assay System (Promega, Madison, WI). For in vivo infection, hCD4/R5/cT1 mice were intrasplenicly or intravenously injected with NL-LucR.T2A-BaL.ecto (1- to 2×10^7^ IU) and evaluated for HIV-1 infection at the indicated times by quantification of LucR activity in splenic lysates as described above. Infection of small intestine and lung was determined by quantifying LucR activity in small intestine segments and lung lysates after enzymatic digestion of the tissue with collagenase D (60 µg/ml) and DNaseI (10 U/ml) as described [22], [23]. Mice were vaginally infected by atraumatically introducing HIV-1-LucR (50 µl containing ∼10^5^ IU) into the vagina 5 days after subcutaneous injection with Depo-Provera (2.5 mg). One week later, vaginal mucosal leukocytes were isolated as described [24], [25] and LucR activity in the cellular lysate was determined. For bioluminescent imaging, the bioluminescence substrate RediJect Coelenterazine h (Caliper Life Sciences, Hopkinton, MA) was directly injected (5 µg) into the spleens prior to ex vivo imaging by the IVIS Spectrum imager (Caliper LifeSciences) using the Wizard bioluminescent selection tool for automatic wavelength and exposure detection. The bioluminescent and gray-scale images were overlaid using the LivingImage 4.0 software package and a pseudocolor image was created representing bioluminescence intensity and quantified as photon counts/second.

### Treatment of Mice with Highly Active Antiretroviral Therapy (HAART) or VRCO1

Mice were treated with HAART by the addition of azidothymidine, lamivudine and indinavir (45 µg/kg/day) to their drinking water as described [26]. Plasma viral load in the mice was determined with a highly sensitive internally controlled real-time reverse transcriptase-initiated quantitative PCR assay that quantifies HIV-1 RNA concentrations down to 1 copy/ml of plasma using gag-specific primers and an internal probe containing a reporter 6-carboxyfluorescein group (FAM) and a 6-carboxytetramethylrhodamine group quencher (Q) as described [27]. VRC01 antibody used for treatment was generated by transfection of CHO cells with a construct expressing VRC01 [28], [29] (provided by Dr. John Mascola, VRC/NIAID/NIH) and passage of the culture supernatant through a protein G column.

### Molecular Engineering of B Cells to Express VRC01 Antibody

We used an approach we previously described [30] to utilize PCR to clone the mRNA sequences encoding the light and heavy chain genes for the secreted VRC01 antibody [28] as a single construct linked by a “self-cleaving” P2A peptide [31] and insert it into the human phosphoglycerate kinase (hPGK) promoter-regulated hPGK.ires.emcvwt.eGFP.Wpre lentiviral transfer vector [32]. Pseudotyped HIV-based third-generation lentivirus was generated by calcium phosphate-mediated co-transfection of 293T cells with the VRC01 or control lentiviral transfer vector and vectors expressing packaging proteins, the rev gene and the VSV-g envelope gene as described [33]. Highly purified mouse B cells isolated from hCD4/R5/cT1 mouse spleens by immunomagnetic sorting using MACS MicroBeads coupled to anti-mouse CD19 and passage through the AutoMACS automated were activated with LPS (50 µg/ml) and one day later were transduced with the VRC01 or control lentivirus (100 ng of p24 antigen, ∼10^8^ transducing units/ml) as described [34]. Transduced or untransduced mouse B cells (5×10^6^ cells) were then intrasplenicly injected into the hCD4/R5/cT1 mice three days after LPS-activation. Two days later the mice were intravenously injected with NL-LucR.T2A-BaL.ecto (1-to 2×10^7^ IU). Seven days later, the mice were bled and serum levels of VRC01 were measured by ELISA as described, [28] and systemic HIV-1 infection was quantified by measuring LucR activity in splenic lysates.

### Ethics Statement

This study was carried out in strict accordance with the recommendations in the Guide for the Care and Use of Laboratory Animals of the National Institutes of Health under a protocol approved by the Einstein Institutional Animal Care and Use Committee (Protocol number: 20101212). All surgery was performed under sodium pentobarbital anesthesia, and all efforts were made to minimize suffering.

## Results

### Construction of Transgenic Mice With Targeted Expression of Human CD4, CCR5 and Cyclin T1 Expression Targeted to CD4+ T Cells and Myeloid-Lineage Cells

**Figure 2:**
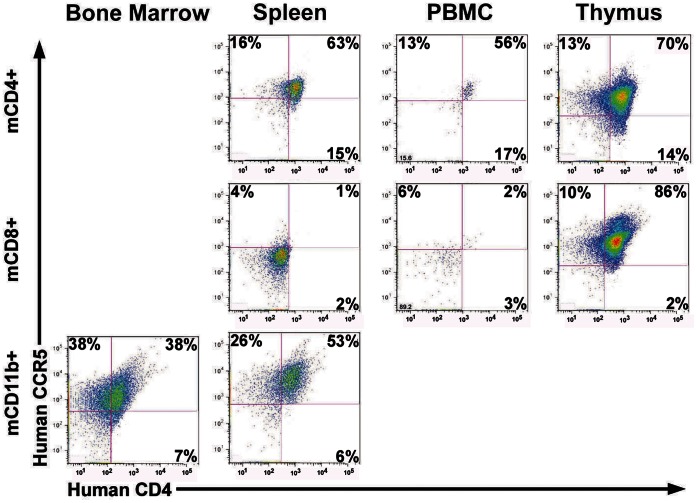
hCD4/R5/cT1 mouse CD4+ T cells and CD11b+ myeloid-lineage cells specifically express human CD4 and CCR5. hCD4/R5/cT1 mouse mononuclear cells isolated from bone marrow, spleen peripheral blood and thymus were evaluated for expression of human CD4 and CCR5 by flow cytometry after staining with antibodies to mouse CD4, CD8 and CD11b and human CD4 and CCR5. Dot plots of human CD4 and CCR5 staining by hCD4/R5/cT1 mouse mononuclear cells gated for the expression of mouse CD4 (upper panels), mouse CD8 (middle panels) or mouse CD11b (lower panels) are shown with the percentage of positive cells in each quadrant indicated.

We used two vectors to generate a mouse line transgenic for the tightly linked transmission of human CD4, CCR5 and cyclin T1 under the control of a CD4 promoter/enhancer that preferentially targets the expression of the transgenes to CD4 T cells and myeloid-lineage cells (Figure 1A) [11], . The first vector was designed to express equimolar quantities of human CD4 and human CCR5 proteins by using a “self-cleaving” picornavirus-like 2A peptide to link these two genes as a single transcript, an approach we previously used to express two genes from a single promoter [13]. The 2A peptide mediates an intraribosomal termination-and-restart event during ribosomal translation of polypeptides which prevents formation of a peptide bond between the two polypeptides it links and thereby produces two separate proteins [35]. Co-injecting this construct into fertilized mouse eggs along with a human cyclin T1 expression plasmid regulated by the same CD4 promoter/enhancer [16], [36] resulted in successful tandem integration and the generation of some founder mice that transmitted these transgenes as a single allele (Figure 1B) [37]. Progeny of a founder mouse selected for breeding expressed human cyclinT1 transgene as determined by immunoblot of their splenocytes and thymocytes (Figure 1C) and human CD4 and CCR5 as determined by flow cytometric analysis of their PBMC and splenocytes (Figure 2). Targeted transgene expression by the CD4 promoter/enhancer to CD4+ T cells and myeloid-lineage cells was demonstrated by the expression of human CD4 and human CCR5 by the majority of the CD4+ T cells in the spleen and peripheral blood as compared to less than 3% of the CD8+ T cells and by the CD11b+ myeloid-lineage cells from the spleen and bone marrow; human CD4 and CCR5 were co-expressed in the CD4^+^ CD8^+^ double positive thymocyte population indicating that expression of human CD4 and CCR5 did not adversely affect thymocyte maturation (Figure 2).

### Productive In Vitro Infection of hCD4/R5/cT1 Mouse CD4+ T Cells and Myeloid-Lineage Cells With HIV-1

**Figure 3:**
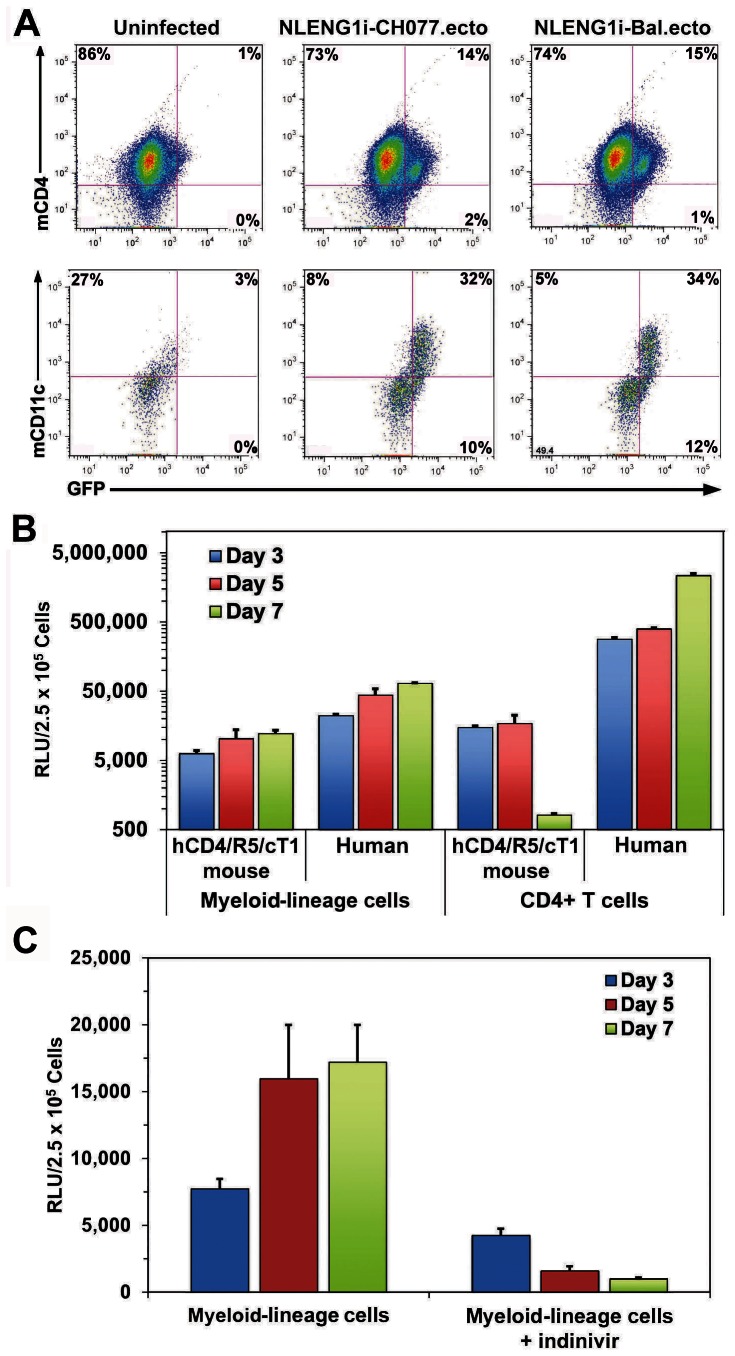
hCD4/R5/cT1 mouse CD4+ T cells and CD11b+ myeloid-lineage cells are susceptible to HIV-1 infection. (**A**) Anti-CD3/CD28-stimulated hCD4/R5/CT1 mouse CD4 T lymphocytes and GM-CSF-stimulated hCD4/R5/cT1 mouse bone marrow-derived myeloid-lineage cells were plated in 48 well plates (1×10^5^ cells/well) and inoculated with NLEng1i-CH077.ecto or NLEng1i-Bal.ecto. After 3 days of culture, HIV-1-infected CD4+T cells (upper panels) and CD11c+ myeloid-lineage cells (lower panels) were quantified by flow cytometry based on their expression of GFP. (**B**) Anti-CD3/CD28-stimulated hCD4/R5/CT1 mouse and human CD4 T lymphocytes and GM-CSF-stimulated hCD4/R5/cT1 mouse bone marrow-derived myeloid lineage-committed cells and human monocytes were infected with NL-LucR.T2A-BaL.ecto. The cells from triplicate or duplicate cultures were harvested on day 3, 5 and 7 and the average LucR activity in the cell lysates ± STE is shown. (**C**) GM-CSF-stimulated hCD4/R5/cT1 myeloid lineage-committed cells were either untreated or treated with indinivir and infected with NL-LucR T2A-BaL.ecto. The cells from duplicate cultures were harvested on day 3, 5 and 7 and the average LucR activity in the cell lysates ± STE is shown.

We examined whether human CD4, CCR5 and cyclin T1 expression by hCD4/R5/cT1 mouse CD4+ T cells and myeloid-lineage cells made them susceptible to HIV-1 infection by infecting them with HIV-1 infectious molecular clones expressing a GFP reporter gene and an Env gene derived either from an R5-tropic isolate, NLENG1i-BaL.ecto, or a R5-tropic transmitted/founder virus, NLENG1i-CH077.ecto [12] and then analyzing them for GFP expression by flow cytometry. Based on our previous demonstration that GM-CSF markedly increased HIV-1 production by myeloid-lineage cells from JR-CSF/hCycT1 mice which carry as transgenes a full-length infectious JR-CSF provirus and a CD4 promoter/enhancer-regulated human cyclin T1 construct [16], we postulated that GM-CSF treatment would also facilitate productive HIV-1 infection of hCD4/R5/cT1 mouse myeloid-lineage cells. Three days after hCD4/R5/cT1 mouse cells were infected with NLENG1i-BaL.ecto or NLENG1i-CH077.ecto, analysis by flow cytometry demonstrated that about 75% of GM-CSF-treated CD11c+ myeloid-lineage cells and 20% of the activated CD4+ T cells were infected by HIV-1 (Figure 3A). This indicated that while expression of the human CD4, CCR5 and cyclin T1 transgenes enabled the hCD4/R5/cT1 mouse CD4+ T cells and myeloid-lineage cells to be infected with HIV-1, hCD4/R5/cT1 mouse myeloid-lineage cells supported infection more robustly than the CD4+ T cells.

*Renilla reniformis* luciferase has a short cellular half-life of approximately 3 hours [38] and LucR continues to be expressed over multiple cycles of replication after inoculation. Consequently, infection with the replication-competent molecular clone, NL-LucR.T2A-BaL.ecto, which was engineered to express the LucR reporter gene and the heterologous BaL *env* gene in *cis* with all of the HIV-1 open reading frames, permits highly sensitive and specific detection of active HIV-1 replication for several weeks after inoculation [12]. Therefore, we infected CD4+ T cells and myeloid-lineage cells from CD4/R5/cT1 mice and humans with NL-LucR.T2A-BaL.ecto and compared their relative capacity to support productive in vitro HIV-1 infection. Activated CD4+ T cells and GM-CSF-treated myeloid-lineage cells from hCD4/R5/cT1 mice, wild-type mice, and HIV-1-naïve human volunteers were infected with NL-LucR.T2A-BaL.ecto and LucR activity was measured in the cellular lysates 3, 5 and 7 days later. GM-CSF-treated hCD4/R5/cT1 mouse myeloid-lineage cells developed sustained HIV-1 infection as evidenced by the 2-fold increase in LucR activity detected over 7 days of culture at levels 20–25% of the 3-fold increase in LucR activity observed for GM-CSF-stimulated human monocytes, while the hCD4/R5/cT1 mouse CD4 T cells supported only transient HIV-1 infection at ∼5% of the levels observed after infection of human CD4 T cells (Figure 3B). The reduced capacity of hCD4/R5/cT1 mouse CD4+ T cells relative to the mouse myeloid-lineage cells to support productive HIV-1 infection is likely due to additional HIV-1 replication blocks reported in mouse T cells [11]. Treatment of the NL-LucR.T2A-BaL.ecto-infected hCD4/R5/cT1 mouse myeloid-lineage cells with the HIV-1 protease inhibitor indinivir at doses that inhibit HIV-1 replication in macrophages [39] significantly reduced LucR levels by greater than 95% (p<.01) (Figure 3C), indicating that cellular LucR levels correlated with productive infection of the cells.

### Productive Systemic In Vivo Infection of hCD4/R5/cT1 Mice With HIV-1

**Figure 4:**
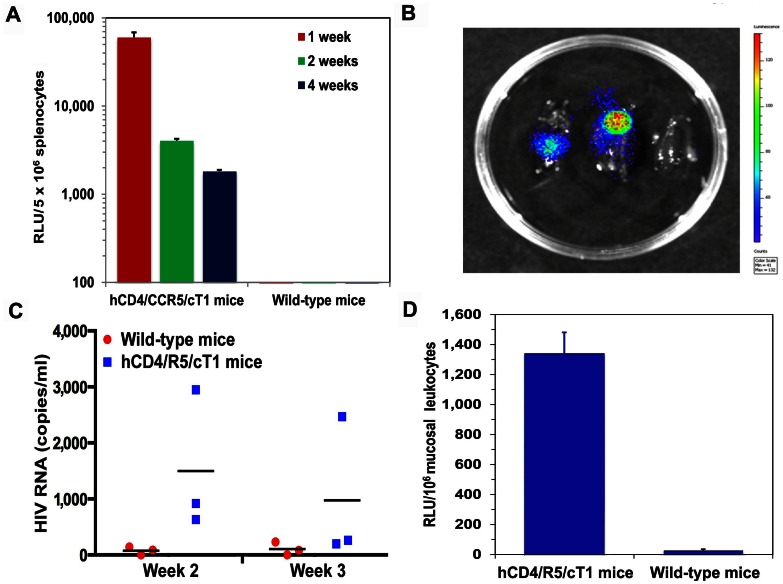
In vivo HIV-1 infection of hCD4/R5/cT1 mice. (A) hCD4/R5/cT1 mice and wild-type mice were intrasplenicly injected with NL4-LucR.T2A-Bal.ecto. LucR activity in the splenic lysates from the mice was measured at 1 week, 2 weeks and 4 weeks (n = 5–8 mice/group) after infection. For all experiments, the average LucR activity in the spleens of the mice in each group ± STE is shown. (B) hCD4/R5/cT1 mice (n = 2) or a control C57BL/6 mouse were intrasplenicly injected with NL4-LucR.T2A-Bal.ecto and 7 days later the spleens were harvested. After ex vivo injection with RediJect Coelenterazine h, bioluminescent and grey-scale images of isolated spleens from the infected hCD4/R5/cT1 mouse spleens (left and center) and the control C57BL/6 mouse (right) were captured with the IVS Spectrum imager and bioluminescence intensity represented in a pseudocolor image indicating photon counts/second are shown. (C) HIV-1 RNA levels in the plasma of hCD4/R5/cT1 and wild-type mice (n = 3 mice/group) were quantified at 2 and 3 weeks after intrasplenic injection with NL4-LucR.T2A-Bal.ecto. (D) Five days after hCD4/R5/CT1 mice or wild-type mice were intravaginally challenged with NL4-LucR.T2A-Bal.ecto (n = 5 mice/group), the LucR levels in leukocytes isolated from the vaginal tissues were determined.

The development of immunocompetent mice that develop systemic infection after exposure to HIV-1, would greatly advance the development and testing of HIV-1 therapies and vaccines. To determine whether the hCD4/R5/cT1 mice developed in vivo infection, we measured LucR activity in the splenocytes 1, 2 and 4 weeks after intrasplenic injection of hCD4/R5/cT1 and C57BL/6 mice with NL-LucR.T2A-BaL.ecto. Productive in vivo infection was evident 1 week post-injection as indicated by the detection of significantly higher levels of LucR activity in splenocytes from all of the injected hCD4/R5/cT1 mice as compared to almost no LucR detected in splenocytes from injected C57BL/6 mice (p<.001) (Figure 4A). Diffuse infection of the spleen was demonstrated by visualization of LucR reporter gene expression by ex vivo imaging of bioluminescent signals in the spleens of the two NL-LucR-T2A-Bal.ecto-injected hCD4/R5/cT1 mice compared to no bioluminescence detected in the spleen from a NL-LucR-T2A-Bal.ecto-injected C57BL/6 mouse (Figure 4B). Further evidence for the development of productive infection was indicated by the detection of a low level of plasma viremia in all of the hCD4/R5/cT1 mice 1 week after infection with NL-LucR-T2A-Bal.ecto (Figure 4C). While the levels of splenic LucR activity and plasma viremia in the hCD4/R5/cT1 mice decreased by 2 to 3 weeks after inoculation, a low level of persistent infection in the spleens continued to be detected 4 weeks after inoculation (Figure 4A).

The mucosal route is the most common site for the acquisition of HIV infection. Therefore, we examined the susceptibility of these mice to HIV-1 acquisition by the vaginal mucosal route. NL-LucR-T2A-Bal.ecto was atraumatically introduced into the vaginas of Depo-Provera-treated hCD4/R5/cT1 and wild-type mice. Five days later, HIV-1 infection was determined by quantifying the level of LucR activity present in vaginal mucosal leukocytes isolated from the vaginal tissue as described [24], [25]. While no LucR was detected in mucosal leukocytes from the wild-type mice, significantly higher levels (p<.01) of LucR activity were detected in mucosal leukocytes isolated from all of the inoculated hCD4/R5/cT1 mice. This indicated that efficient HIV-1 acquisition across the mucosal route associated with the initiation of localized HIV-1 infection occurred in the hCD4/R5/cT1 mice (Figure 4D).

**Figure 5:**
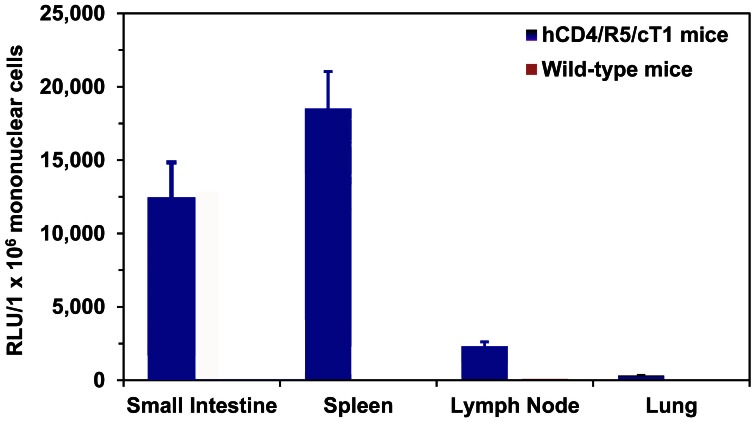
hCD4/R5/cT1 mice develop disseminated HIV infection after intravenous injection. hCD4/R5/cT1 mice and wild type mice (n = 5 mice/group) were intravenously injected with NL4-LucR.T2A-Bal.ecto. One week later, mononuclear cells were harvested from the mouse spleens, small intestines, iliac lymph nodes and lungs. The average LucR activity in the cellular lysates from the indicated tissues of the hCD4/R5/cT1 mice and wild-type mice in each group ± STE is shown.

The intravenous route of HIV-1 infection through drug injection with contaminated syringes is a major risk factor for the acquisition and transmission of HIV, accounting for about 5–10% of HIV infections worldwide [40]. We therefore examined whether the hCD4/R5/cT1 mice could also be infected by intravenous exposure and used to study HIV transmission by the intravenous route and the role of substance abuse in facilitating HIV-1 acquisition. One week after intravenous injection of hCD4/R5/cT1 and wild-type mice with NL-LucR-T2A-Bal.ecto, mononuclear cells were isolated from the mouse spleens, small intestine, lymph node and lungs and examined for luciferase activity as a proxy for quantifying HIV infection. After intravenous injection of NL-LucR-T2A-Bal.ecto, disseminated productive HIV-1 infection, as indicated by the detection of LucR, developed in the mononuclear cells isolated from the mouse spleens, small intestine, lymph nodes and lungs of all of the hCD4/R5/cT1 mice compared to no infection in the wild-type mice (p<.0001) (Figure 5). The higher level of HIV-1 infection detected in the spleens compared to the lymph nodes may be due to the efficient delivery of lentiviruses into spleens after intravenous injection [41], [42] or alternatively the level of infection in the iliac lymph nodes we sampled may not be indicative of the infection levels in other lymph nodes.

### hCD4/R5/cT1 Mice can be Used to Evaluate the Therapeutic Efficacy of Antiretroviral Therapy and of Broadly Neutralizing Antibodies Delivered Either Passively or By Gene Therapy

**Figure 6:**
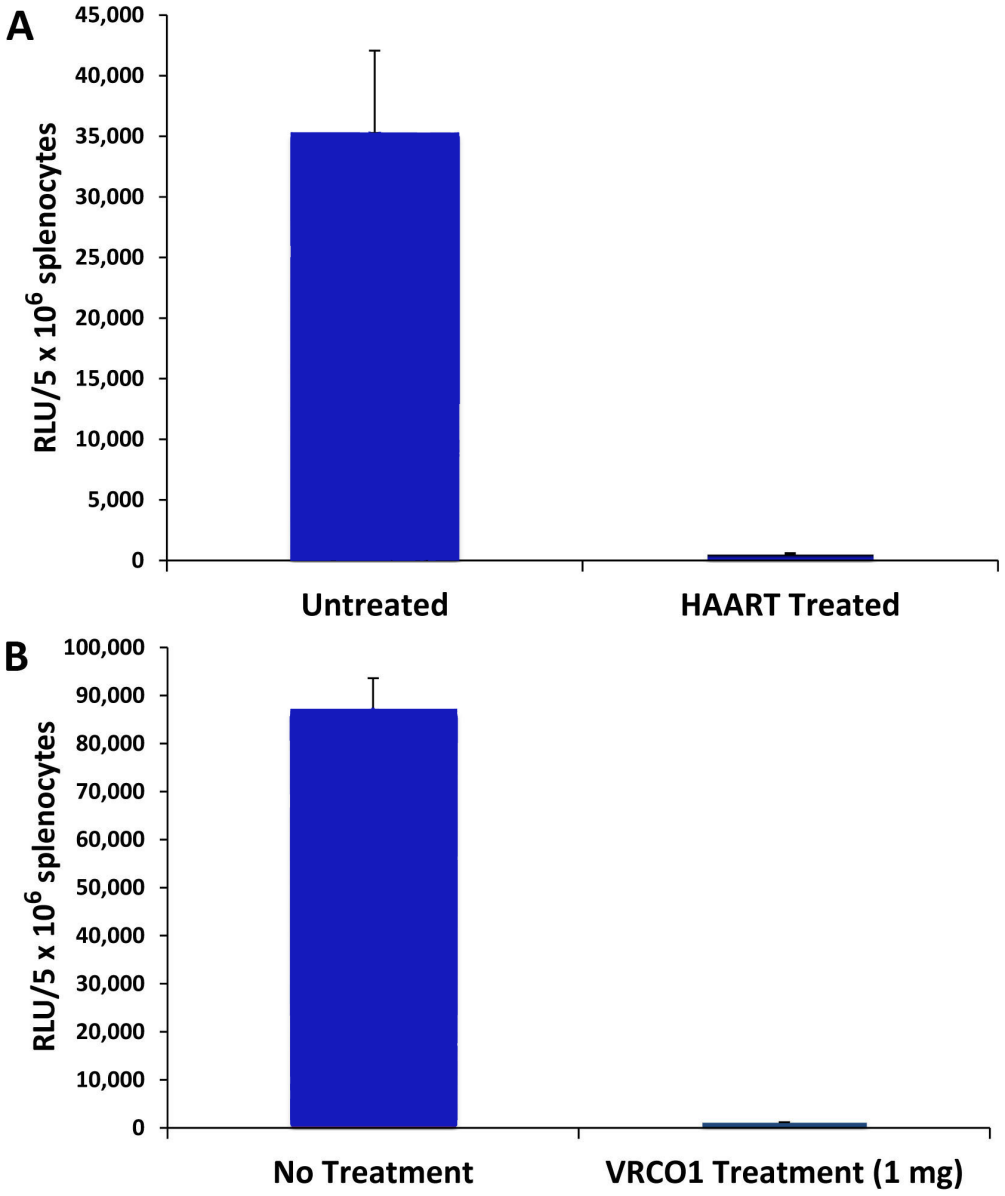
In vivo HIV-1 infection of hCD4/R5/cT1 mice is inhibited by antiretroviral therapy or by treatment with a broadly neutralizing antibody. (A) One group of hCD4/R5/cT1 mice (n = 4 mice) was untreated and another group (n = 4 mice) was started on HAART administered in their drinking water. Five days later these mice were infected with NL4-LucR.T2A-Bal.ecto by intrasplenic injection. (B) hCD4/R5/cT1 mice were untreated (n = 5 mice) or treated with one intravenous dose (1 mg) of VRC01 (n = 5 mice). The next day, the mice were infected with NL4-LucR.T2A-Bal.ecto by intrasplenic injection. One week later, HIV-1 infection was quantified by measuring LucR activity in the splenic lysates. The average LucR activity for the mice in each group +/− STE is shown.

We investigated whether the extensive HIV-1 infection detected in all of the hCD4/R5/cT1 mouse spleens one week after injection with NL-LucR-T2A-Bal.ecto would enable us to use this mouse as a model to evaluate the efficacy of anti-retroviral therapies. Untreated hCD4/R5/cT1 mice or hCD4/R5/cT1 mice treated with HAART by oral administration of azidothymidine, lamivudine and indinivir [26] for 5 days were intrasplenicly injected with NL-Luc-T2A-BaL.ecto. One week later, the level of HIV-1 replication in their spleens was measured by quantification of LucR activity in splenic lysates. HIV-1 infection was significantly inhibited by almost 99% in all of the hCD4/R5/cT1 mice treated with HAART, as compared to the level of HIV-1 infection in the untreated hCD4/R5/cT1 mice (p<.01) (Figure 6A).

The use of broadly neutralizing anti-HIV antibodies as a therapeutic modality to treat HIV-1-infected individuals has been supported by a recent study that demonstrated that combinations of potent neutralizing antibodies can control in vivo HIV-1 infection in humanized mice [41]. A transgenic mouse model supporting acute HIV-1 infection does not require access to human hematopoietic stem cells and the individualized engraftment procedures that are required to produce humanized mice. The increased accessibility of this transgenic mouse model would greatly facilitate further studies to optimize the application of this therapeutic approach by determining the bioavailability and synergistic activity of various broadly neutralizing anti-HIV antibodies. Therefore, we examined whether the hCD4/R5/cT1 mice could be used to evaluate the in vivo capacity of broadly neutralizing anti-HIV-1 antibodies such as VRC01 [28], [29] to inhibit HIV-1 infection. One group of hCD4/R5/cT1 mice was intravenously injected with VRC01 (1 mg). This dosage was used because we determined that one week after injection this dose provides an average serum level of VRC01 of ∼60 µg/ml; this concentration is greater than the 50% inhibitory concentration for neutralization of about 90% of major circulating HIV-1 clades [28]. The next day, the VRC01-treated and control untreated hCD4/R5/cT1 mice were intrasplenicly injected with NL-LucR.T2A-BaL.ecto and one week later the mice were sacrificed and LucR activity in their spleens was determined. Splenic lysates from hCD4/R5/cT1 mice treated with VRC01 displayed 95% lower levels (p<.05) of LucR activity as compared to splenic lysates from untreated hCD4/R5/cT1 mice (Figure 6B), indicating that VRC01 potently inhibited in vivo HIV-1 infection.

**Figure 7:**
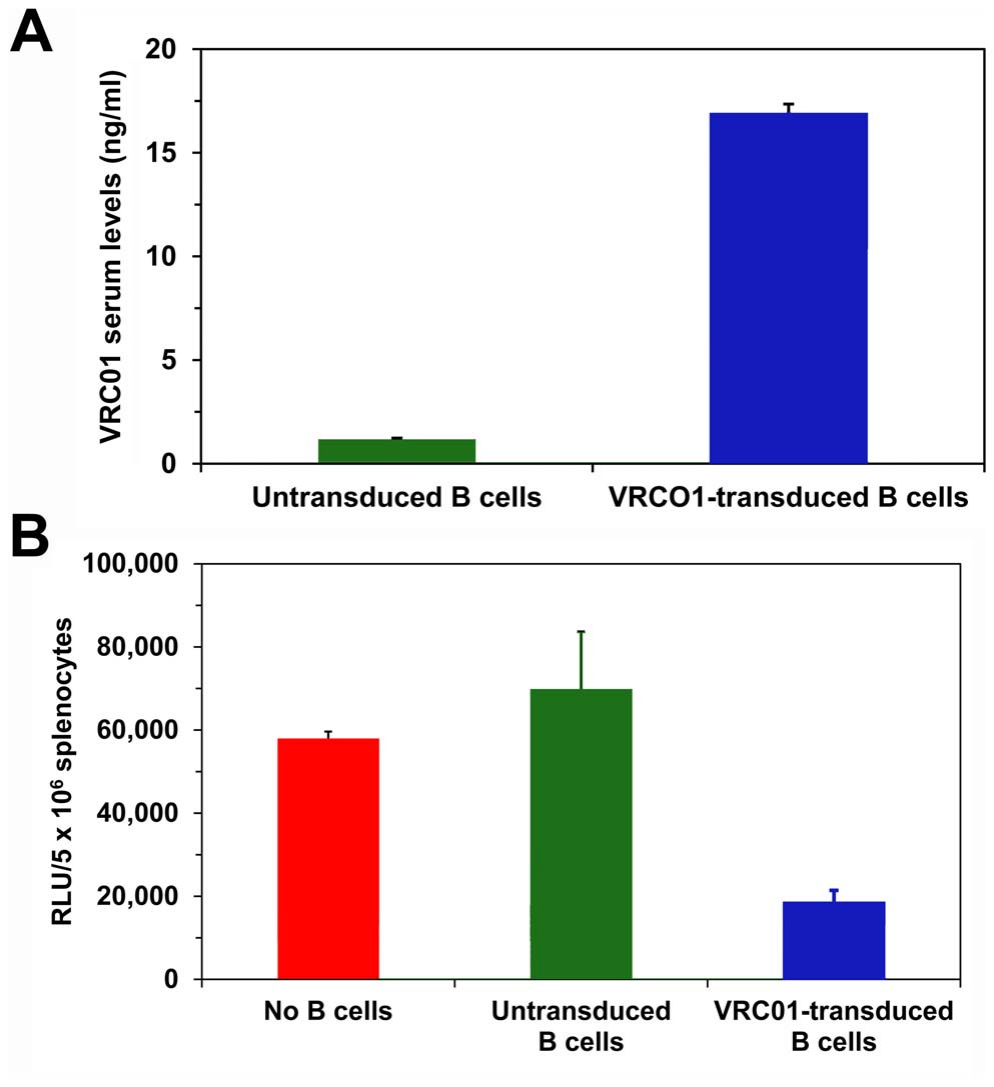
HIV-1 infection of hCD4/R5/cT1 mice is inhibited by VRC01 antibody secreted in vivo by transduced B cells. hCD4/R5/cT1 mice were uninjected (n = 4 mice) or intrasplenicly injected with highly purified primary B cells which were either untransduced (n = 4 mice) or transduced with the VRC01-expressing lentivirus (n = 4 mice). Two days later, the mice were all inoculated intravenously with NL-LucR-T2A-Bal.ecto. Seven days later, the mice were sacrificed. (**A**) The level of VRC01 antibody in the serum was determined by an ELISA assay (**B**) The LucR activity in the mouse spleens was quantified. The average VRC01 serum antibody levels and LucR activity in the spleens for the mice in each group +/− STE is shown.

Molecular delivery of broadly neutralizing antibodies by genetically engineering cells to secrete the antibodies in vivo is a potential therapeutic approach to complement and/or substitute for anti-retroviral treatment with drugs. We have previously demonstrated that we could utilize genetic engineering to efficiently transduce and program primary human B cells to secrete potent neutralizing antibodies that markedly inhibited infection of humanized NOD/SCID/γ_c_^null^ mice [30]. Humanized mice were also protected from HIV-1 infection by intramuscular injection of adeno-associated virus vectors encoding broadly neutralizing antibodies which were secreted into the circulation [42]. Therefore, we examined whether the hCD4/R5/cT1 mice could be used as a model to evaluate the capacity of gene therapeutic approaches to deliver neutralizing HIV antibodies in vivo and prevent HIV infection. Highly purified B cells isolated from hCD4/R5/cT1 mouse spleens were untransduced or transduced with a lentivirus expressing VRC01 and intrasplenicly injected into hCD4/R5/cT1 mice. Two days later, hCD4/R5/cT1 mice which had either not been injected with B cells or had been intrasplenicly injected with untransduced B cells or transduced B cells were inoculated intravenously with NL-LucR-T2A-Bal.ecto. Seven days later, the mice were sacrificed and the serum was analyzed for VRC01 levels and the spleens were evaluated for LucR activity. In vivo production of VRC01 by the transduced B cells was demonstrated by the detection of VRC01 antibody in the serum of the mice intrasplenicly injected with VRC01 lentivirus-transduced B cells and not in the serum of mice intrasplenicly injected with untransduced B cells (Figure 7A). The endogenously produced VRC01 inhibited in vivo infection as evidenced by the significant reduction by about 70% in LucR levels in the spleens of mice intrasplenicly injected with VRC01 lentivirus-transduced B cells compared to mice either not injected with B cells or intrasplenicly injected with untransduced B cells (Figure 7B). Thus, the hCD4/R5/cT1 mice can be used as a model to study the in vivo efficacy of gene therapy approaches to deliver broadly neutralizing HIV-1 antibodies as a treatment modality to prevent HIV-1 infection.

## Discussion

One of the major challenges limiting the evaluation and testing of new HIV-1 therapeutics is the availability of an immunocompetent mouse model that is infectible with HIV-1 without the need for transplantation with human tissues. The restricted availability and significant expense associated with infection studies in macaques as well as biological differences between the SIV or SHIV used to infect macaques and HIV, limits their widespread use as an in vivo model to study the efficacy of new therapeutic approaches to treat or prevent HIV-1 infection. While humanized mice constructed by engrafting highly immunodeficient mice with human hematopoietic cells and or tissues overcome some of these limitations, their use is limited by the specialized facilities and techniques required for their construction and housing. Furthermore, the humanized mice display a high degree of mouse-to-mouse variation due to variable levels of engraftment and the heterogeneity of donor tissue and the immune system developed by these mice is suboptimal, precluding their use in vaccine studies. In addition, the immune cells in the humanized mice are human and therefore are not subject to genetic manipulation for experiments using transgenic and knockout mice. A highly creative mouse model that was recently described used transgenic mice carrying an inducible loxP-STOP-loxP luciferase reporter whose expression can be triggered after infection with pseudotyped HIV-1 that expresses Cre recombinase; hepatocytes from these mice are rendered susceptible to HIV entry by infection with intravenously injected recombinant human adenovirus serotype 5 encoding the human CD4 and CCR5 genes [43]. While useful for studying antibody-mediated inhibition of entry, this is a complex model which only supports HIV-1 entry and not HIV-1 replication and whose target cells, hepatocytes, are not normally a target for HIV-1 infection.

In the current study, we reported on the development of a new fully transgenic mouse model, hCD4/R5/cT1 mice, which supports in vivo replication of HIV-1 in the appropriate target cells for HIV-1 infection, CD4+ T cells and macrophages. Furthermore, we demonstrated the application of hCD4/R5/cT1 mice for studying HIV-1 acquisition and the in vivo efficacy of HIV-1 therapeutics. We developed this model by using a novel strategy of constructing triple-transgenic mice which efficiently transmit human CD4, CCR5 and cyclin T1 transgenes as a single allele and infecting the mice with replication-competent HIV-1 infectious molecular clones expressing LucR. Infection of the hCD4/R5/cT1 mice with the HIV-1 infectious molecular clone expressing LucR enabled the mice to be used as an in vivo system to detect with high sensitivity and specificity the acquisition of HIV-1 infection and to evaluate the in vivo capacity of antiretroviral therapy and broadly neutralizing anti-HIV-1 antibodies to prevent infection. Although the hCD4/R5/cT1 mice did not display continued expansion of the acute HIV-1 infection, all of the inoculated mice reproducibly developed substantial primary infection which continued to increase for the first week after intrasplenic or intravenous inoculation. Furthermore, HIV-1 infection occurred in the mucosal tissues of all of the mice inoculated by the vaginal route. This susceptibility to acute HIV-1 infection should enable these mice to be used as a new pre-clinical in vivo model to evaluate the capacity of therapeutics or immune responses such as neutralizing antibodies to block acute HIV-1 infection. In contrast to humanized mice constructed by engrafting immunodeficient mice with human hematopoietic stem cells, these mice have a fully functional murine immune response. Therefore, the hCD4/R5/cT1 mouse model should be useful to evaluate the capacity of HIV-1 vaccines to induce protective humoral and cellular adaptive immune responses. These potential applications will also greatly benefit from using the LucR-expressing infectious molecular clones as the challenge inoculum because the NL-Luc-proviral backbone-based infectious molecular clone has been engineered to facilitate the expression of a diverse range of HIV-1 env sequences [12]. LucR-expressing infectious molecular clones have been constructed that express over 60 Envs from different clades, including clade A/E and C Envs and transmitted/founder Envs which are relevant for transmission studies and vaccine trials. Infection by these infectious molecular clones that express diverse Envs from clinically-relevant isolates will be particularly useful for applying this mouse model to evaluate the in vivo antiviral efficacy of broadly neutralizing anti-HIV-1 antibodies and the protective capacity of antibodies induced by candidate HIV-1 vaccines to prevent intravenous and mucosal acquisition of HIV-1 infection. Because these mice are genetically identical and do not require the time, effort, expertise, and access to human fetal tissue or cord blood required for constructing humanized mice [5]–[8], [10], they should provide an new in vivo infection model that is highly reproducible, inexpensive and widely available for the HIV-1 research community.

While the hCD4/R5/cT1 mice should be an excellent in vivo model for evaluating the capacity of therapeutics and the adaptive immune response to prevent HIV-1 infection, a limitation of this model is that the level of HIV-1 infection in the hCD4/R5/CT1 mice waned and did not increase after the initial establishment of disseminated infection. This was likely a result of the reduced capacity of the hCD4/R5/CT1 mouse CD4+ T cells to support HIV-1 replication due to in vitro post-transcriptional defects in Gag expression, processing and release that severely reduce HIV-1 replication in mouse T cells compared to mouse macrophages [11], [36]. This was supported by our in vitro findings that hCD4/R5/cT1 CD4+ T cells developed markedly lower and less sustained in vitro HIV-1 infection as compared to hCD4/R5/cT1 myeloid-lineage cells or primary human CD4 T cells. The increased HIV-1 production by infected hCD4/R5/cT1 mouse macrophages we observed as compared to hCD4/R5/cT1 mouse T cells may be due to the capacity of macrophages to utilize a different HIV-1 assembly pathway than T cells [44]. HIV-1 assembly in T cells occurs at the plasma membrane [45], while HIV-1 assembly in macrophages can occur in the late endosomal compartment, particularly the major histocompatibility class II compartment [46]. In vitro GM-CSF-treatment may enable hCD4/R5/cT1 mouse myeloid-lineage cells to support in vitro HIV-1 infection at almost 25% of the levels observed after infection of primary human monocyte/macrophages by stimulating the production of multivesicular bodies, a well-described site for Gag assembly and HIV-1 budding from infected cells [47] and thereby circumventing the plasma-membrane-associated blocks that compromise Gag processing and T cell infection. The persistent in vivo infection we observed in mouse spleens one month after inoculation (Figure 4A), albeit at lower levels than 1 week after inoculation, may represent sustained in vivo replication in myeloid-lineage cells.

To improve the model and particularly to enable systemic HIV-1 infection after mucosal exposure, we are endeavoring to increase the capacity of mouse CD4+ T cells to support HIV-1 replication by identifying the human genes required to overcome other species-specific blocks to HIV-1 replication which could be expressed as transgenes in hCD4/R5/cT1 mice. An alternative approach we are pursuing is to generate HIV-1 variants with an increased capacity to replicate in hCD4/R5/cT1 mice. We have crossed the hCD4/R5/cT1 mice with our JR-CSF/hu-CycT1 mice [16] to generate mice whose CD4 T cells and myeloid-lineage committed cells express the JR-CSF proviral transgene and human CD4, CCR5 and cyclin T1. Support of the full HIV-1 replication cycle including the error-prone step of reverse transcription by these mice, should populate the mice with a swarm of randomly generated and sequentially divergent HIV quasipecies which over time should undergo in vivo selection for their increased capacity to replicate in mouse cells as described for the in vivo selection of drug-resistant and immune-evasion isolates [48]. Identification of mutations that increase the capacity of HIV-1 to replicate in mouse cells will enable us to genetically alter NL-LucR.T2A-BaL.ecto to increase its capacity to replicate in mouse cells. Infection with this modified NL-LucR.T2A-BaL.ecto may support the development of sustained infection in hCD4/R5/cT1 mice and/or systemic infection after mucosal exposure, further enhancing the utility of the hCD4/R5/cT1 mice for evaluating the efficacy of anti-HIV-1 therapeutics and vaccines.
